# Reliability and Concurrent Validity of Global Physical Activity Questionnaire (GPAQ): A Systematic Review

**DOI:** 10.3390/ijerph16214128

**Published:** 2019-10-26

**Authors:** Xiaofen D. Keating, Ke Zhou, Xiaolu Liu, Michael Hodges, Jingwen Liu, Jianmin Guan, Ashley Phelps, Jose Castro-Piñero

**Affiliations:** 1 Department of Curriculum and Instruction, The University of Texas at Austin, Austin, TX 78712, USA; xk93@austin.utexas.edu (X.D.K.); Xiaolu.liu@utexas.edu (X.L.); ashley.phelps@utexas.edu (A.P.); 2 Institute of Physical Education, and Bioinformatics Center, Henan University, Kaifeng 475001, China; 3 Department of Kinesiology, William Paterson University, Wayne, NJ 07470, USA; HODGESM1@wpunj.edu; 4 Department of Kinesiology, California State University, Fullerton, CA 92831, USA; jingwliu@fullerton.edu; 5 Department of Health, Kinesiology, and Nutrition, The University of Texas at San Antonio, San Antonio, TX 78249, USA; jianmin.guan@utsa.edu; 6 GALENO research group, Department of Physical Education, Faculty of Education Sciences, University of Cadiz, 11519 Puerto Real, Spain; jose.castro@uca.es

**Keywords:** adult physical activity questionnaire, international perspective, revalidation

## Abstract

This study aimed to systematically review previous studies on the reliability and concurrent validity of the Global Physical Activity Questionnaire (GPAQ). A systematic literature search was conducted (*n* = 26) using the online EBSCOHost databases, PubMed, Web of Science, and Google Scholar up to September 2019. A previously developed coding sheet was used to collect the data. The Modified Quality Assessment Tool for Observational Cohort and Cross-Sectional Studies was employed to assess risk of bias and study quality. It was found that GPAQ was primarily revalidated in adult populations in Asian and European countries. The sample size ranged from 43 to 2657 with a wide age range (i.e., 15–79 years old). Different populations yielded inconsistent results concerning the reliability and validity of the GPAQ. Short term (i.e., one- to two-week interval) and long-term (i.e., two- to three-month apart) test–retest reliability was good to very good. The concurrent validity using accelerometers, pedometers, and physical activity (PA) log was poor to fair. The GPAQ data and accelerometer/pedometer/PA log data were not compared using the same measurements in some validation studies. Studies with more rigorous research designs are needed before any conclusions concerning the concurrent validity of GPAQ can be reached.

## 1. Introduction

Participation in physical activity (PA) on a regular basis is well documented as a critical component of a healthy lifestyle and disease prevention [1]. However, public health organizations, educational institutions, and others interested in an intervention project should have valid and reliable scales for measuring PA [2,3,4]. Without validated PA measurement scales, it is impossible to accurately assess and monitor the progress of PA interventions fairly and in different settings [5,6]. Although technologies such as pedometers and accelerometers have increased the objectivity and accuracy of PA measures [7,8], self-reported survey methods still have the advantage to reach out to a large scale of the general population due to its low costs [5,9]. To date, several different PA questionnaires have been developed and validated for use in developed countries [2,6]. These questionnaires are of limited scope and scale because most place high importance on leisure time PA [1,2], while people participate in PA during work and commute rather than in their leisure-time in many developing countries [9,10,11,12,13,14]. Therefore, there is a need to develop a valid and reliable questionnaire that can accurately measure PA behaviors across countries [2,6].

The Global Physical Activity Questionnaire (GPAQ), a modified version of the International Physical Activity Questionnaire (IPAQ) [6], was developed by the World Health Organization (WHO) in response to a greater interest in the role of PA in health in 2002 [15]. The aim of the GPAQ was to enhance the IPAQ in cross-cultural settings [2,15,16,17]. The GPAQ uses a standardized protocol as surveillance of PA engagement at the population level with self-report and interview-administrated modes [15,18]. The original version of the GPAQ contained 19 questions with a shorter version later developed, eliminating three redundant questions, totaling to 16 questions for the most updated version [6]. The GPAQ has questions revolving around three domains: Occupational, transport-related, and leisure-time PA [1,2,18]. For each domain, there is a pre-set PA list to help participants recall PA, which ensures the reliability and validity of the questionnaire [2,6]. Moreover, the GPAQ can also standardize data collected, focusing on the moderate-to-vigorous PA (MVPA) for work and recreation, minutes of walking and bicycling for transport only [15]. Noticeably, the GPAQ assesses sedentary behavior (SB) by collecting minutes spent in sitting activities [15,19,20]. The GPAQ also uses “a typical week” in its questionnaire about PA that lasts for at least 10 min with the moderate and vigorous intensity [1,2]. As such, the GPAQ is missing an element that accurately measures light PA embedded into work and recreational spaces.

The GPAQ has to be reliable, valid, and adaptable across all populations if it is to monitor PA behaviors and provide global guidelines for future interventions. Hence, many studies have examined its validity and reliability in the past [1,9,12,19,21,22], yet, no systematic reviews have been conducted concerning the reliability and validity of the GPAQ across countries since its inception in 2002. Empirical evidence concerning the validity and reliability of the GPAQ would help us better understand the overall quality of the questionnaire and shed a new light on the GPAQs worldwide feasibility. The proposed systematic review could also provide a basis for comparison studies using GPAQ in future research. It is hoped that the gaps uncovered in our knowledge about the GPAQ found by this review would help identify directions for future research on the topic in different countries. Thus, this paper aimed to systematically review the GPAQs reliability and concurrent validity found in previous studies.

## 2. Materials and Methods

### 2.1. Selection Criteria

A literature search using PubMed, Google Scholar, Web of Science, and EBSCOhost databases was conducted to identify pertinent articles using the following keywords: Validity, concurrent validity, reliability, validation, global physical activity questionnaire, and GPAQ. These databases have been deemed to have the widest coverage of research articles in education, psychology, sports, PA, health, and physical education published in English [3,23,24]. Only peer-reviewed journal articles were selected for review if they fulfilled the following criteria: Written in English, analyzed/discussed the reliability and/or validity of the GPAQ. References cited by the selected articles were also explored to ensure that all eligible publications were included for review. The time frame was set from 2002 at GPAQ’s inception to September 2019. Studies using the GPAQ to collect PA data were excluded. Conference abstracts and papers were also eliminated (see Table 1).

### 2.2. Data Reduction and Harmonization

A coding sheet was first developed based on the purposes of the study and the PRISMA-P checklist [25]. Previously published systematic review studies on similar topics [5,26,27] were also examined to help generate the complete coding sheet. The following data were coded: Countries in which the GPAQ was tested, first author’s name, year of publication, research design, the number of participants, participants’ mean age, gender, ethnicity, and occupation, comparison devices/methods, and values of validity and reliability measure(s).

Using the above pre-established inclusion/exclusion criteria, three independent reviewers with a PA questionnaire validation background initially screened all abstracts of the 391 retrieved articles, rating each abstract as either yes, no, or maybe for inclusion. The ones rated as yes were kept and those rated as no by both reviewers were eliminated. The maybe ones were reviewed by a third reviewer and a consensus was reached through discussion for any disagreement among the three reviewers. A total of 26 articles met the inclusion criteria and were retrieved for complete review and analysis. The entire text of each article evaluated was examined by three investigators. The other two authors served as the reliability coders by independently coding all the 26 articles to ensure the acceptable inter-coder reliability (agreement > 80%) [28]. Discrepancies between coders were discussed among all team members until the consensus was reached.

### 2.3. Methodological Quality and Risk of Bias

The methodological quality and risk of bias of the selected articles were assessed using The Modified Quality Assessment Tool for Observational Cohort and Cross-Sectional Studies [29], based on the purpose of the study and the content domains included, ultimately, allowing us to assess the appropriateness of the methodology, study design, participant selection, and data analysis. The following two modifications were made. First, instead of using a checklist style (i.e., yes or no), a three-point Likert scale (i.e., 3 = good, 2 = fair, and 1 = poor) was employed to evaluate the items included in the aforementioned tool. And second, the content of the assessment tool was also modified according to previous research on PA questionnaire reliability and validity [3,5]. Specifically, item 13 was not applicable to our paper, and therefore, was deleted. The standards used by Chinapaw et al. [3] and Hidding [5] were adopted to assess the methodological quality of test–retest reliability studies regarding the time interval: Between >1 day and <3 months for questionnaires recalling a standard week, which was used in the GPAQ [6].

Two of the researchers independently rated the articles in each of the items listed in the tool. Since there were only 26 articles with specific reliability and/or validity data, the raters coded all studies separately with specific comments on the weaknesses of those studies which were rated poor. The results of these ratings were compared, and discrepancies among raters were discussed until 100% agreement was achieved.

### 2.4. Synthesis of Results

#### 2.4.1. Reliability

The following types of studies were included for synthesis if it reported intraclass correlation coefficients (ICC) for continuous measurements of different raters [30], and/or Pearson r for continuous variable and Spearman’s correlation coefficients (rho) for rank variables, and/or agreement measures using Cohen’s simple kappa (κ) for binary ratings and weighted κ for two inter-rater agreement assigning different weights to the different levels [30,31]. It was noted that a single study may report values of kappa, weighted kappa, and ICC when continuous data are used [32]. According to Warner [31], the cut-off value of r or rho or κ for poor, moderate (acceptable), or strong is <0.4, 0.4–0.8, and >0.8, respectively. For ICC, the cut-off values are slightly different (i.e., poor: ICC < 0.5, moderate: 0.5 < ICC < 0.70, good: 0.70 < ICC < 0.90, excellent: ICC > 0.90). Because the sample size and participant characteristics varied greatly among the selected studies, the mean value of test–retest reliability was not calculated. Instead, the range of reliability and validity was examined.

#### 2.4.2. Concurrent or Criterion Validity

Concurrent validity is defined as the degree to which the measurement values of the GPAQ are consistent with a criterion-related standard [4,20,22,32]. The criterion-related standard was established by both self-reported (i.e., PA log and IPAQ) and objectively measured data (i.e., pedometers and accelerometers). In general, concurrent validity often tested using Spearman’s rho and the Bland–Altman plots for visually assessing the absolute agreement between two different methods when the same variable was measured [28,30]. The mean bias could also be examined using the Bland–Altman plots. The aforementioned cut-off values for rho were used to assess the magnitude of concurrent validity. The concurrent validity of three domains of PA and SB was also synthesized.

## 3. Results

### 3.1. Article Selection

**Figure 1:**
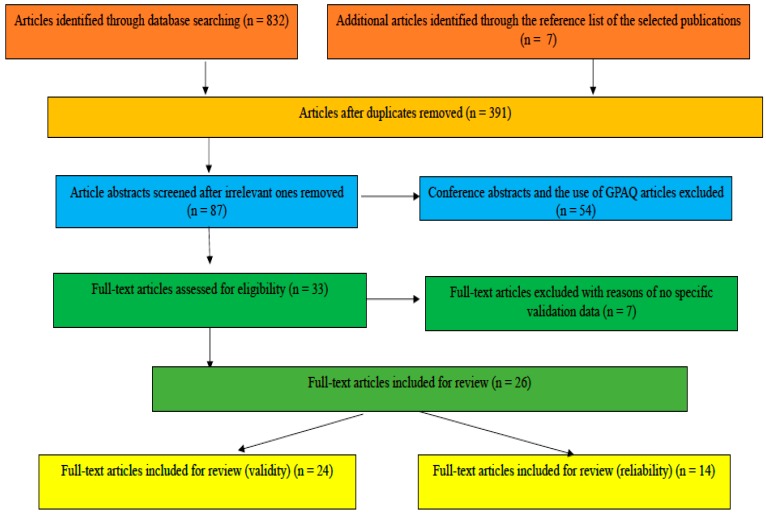
Flow chart of articles searched (Note: Some studies examined both reliability and validity and thereby the total N is greater than 26).

A total of 26 publications were included in the review (see Figure 1). These studies validated the GPAQ in 18 independent countries and two in multiple countries from 2002 to September 2019 [1,7,9,11,12,16,18,19,20,33,34,35,36,37,38,39,40,41,42,43,44,45,46,47,48]. There were five countries (i.e., India [36,46], Malaysia [9,37], Singapore [7,18], US [20,41,42,43], and Vietnam [12,43]) with more than one study on the topic, excluding the ones conducted in multiple countries.

Among these completed in the 18 countries, 10 were in Asian countries (i.e., Bangladesh, China, India, Korea, Malaysia, Saudi Arabia, Singapore, Thailand, The United Arab Emirates (UAE), and Vietnam) with a total of 20 publications (i.e., 76.9%). The rest were from European countries and the US. However, one of the two validation studies was completed in multiple countries reporting the validation data from nine countries [1]. The other was done in Belgium, Spain, and UK [44].

The representation of other continents was limited given that there was one study found in South America [19] and Africa [39], respectively, even though it was reported that the GPAQ has been used in many African countries [1,2]. Moreover, only one article included participants who were younger than 18 [36]. No studies specifically examined the reliability and validity of the GPAQ in the elderly group, even though many studies have included participants whose age was above 60 years old [7,11,12,20,36,40,42,43].

The GPAQ could be administered by others using face-to-face interviews or self-administered [2,18,47,48]. An interviewer-administered GPAQ requires a trained interviewer, while self-administered may be more cost-effective, if valid and reliable. Chu and colleagues [18] tested the differences in using self- and interviewer-administered modes and found a similar level of comparability between the two administrations (see Table 2).

### 3.2. Methodological Quality and Risk of Bias

All studies used a cross-sectional research design (see Table 3). The GPAQ data were compared to accelerometer and/or pedometer data except for one study, which used a PA log [11]. The quality of study scores ranged from 1 to 3 (i.e., the higher the mean, the better study quality). Methodological shortcomings were mostly identified as not wearing pedometers or accelerometers during the typical week in which the GPAQ aimed to measure. A smaller sample size was the second common weakness noted (see Table 4).

### 3.3. Concurrent Validity of GPAQ

A cross-sectional research design was employed by all studies. Most selected studies (24 out of 26) examined the concurrent validity of the GPAQ by comparing the weekly GPAQ data with a criterion measure(s). Eight studies used pedometer and/or a PA log and/or IPAQ data as the criterion measure while the rest of the studies used either accelerometers or pedometers (Note: Some studies used more than one criterion standard) (see Table 5, Table 6 and Table 7). Spearman’s rho was calculated to examine criterion validity. Bland–Altman plots were also used to test the acceptable agreement between the two sets of data.

#### Concurrent Validity Results

As noted earlier, the GPAQ data were compared to either accelerometers and/or pedometers and/or a PA log and/or a previously validated PA questionnaire (e.g., IPAQ). Data collected by ActiGraph GT3X (AG) was most often employed in the validation studies (see Table 5). Criterion validity for the overall PA, the three PA domains, and SB, respectively, was examined. The concurrent validity for work-related PA (*r*: −0.03–0.50), transport-PA (*r*: 0.04–0.49), and leisure-PA (*r*: 0.02–0.41) was poor to fair. So was the SB concurrent validity (*r*: 0.07–0.47). In addition, MVPA concurrent validity using accelerometers was mostly often investigated, followed by moderate PA (MPA) and vigorous PA (VPA). Specifically, MVPA concurrent validity using accelerometers and pedometer, PA log and IPAQ was −0.01–−0.69, and −0.01–−0.54, respectively. In essence, the results of the criterion validity for various GPAQ measures ranged from poor to fair using Spearman’s rho (see Table 5, Table 6 and Table 7).

The concurrent validity results using both pedometers and accelerometers, surprisingly, were about the same (*r* < 0.5), even though accelerometers are found to generate more accurate PA data than pedometers [45]. Moreover, pedometers only measure steps without PA intensity data. However, different total daily steps were used to indirectly measure PA with different intensities. For instance, Sitthipornvorakul and colleagues [32] classified pedometer (steps/day) inactive (<5000), moderately active (5000–9999), and highly active (≥10,000). This was how pedometer data quantified the PA intensity (see Table 6). PA domain-specific concurrent validity of the GPAQ was also poor to fair compared to that measured by accelerometers, pedometers, or a PA log. There was only one study using the IPAQ that found very good concurrent validity of the GPAQ in India [36].

When the GPAQ was analyzed on a country level, each country had a slightly different result. For instance, using the same method by comparing GPAQ data with AG accelerometer data, some researchers reported higher correlations for minutes of MVPA in the US (*r* = 0.26) [42], China (*r* = 0.26–0.52) [11], and the UK (*r* = 0.48) [16] than that found by Bull and colleagues [1] in lower-income countries such as South Africa (*r* = −0.03). Moreover, Bland–Altman plots found that participants tended to overestimate their MVPA and underestimate their SB using the GPAQ.

### 3.4. Test–Retest Reliability of GPAQ

There were 14 studies (i.e., 53.8%) that tested the reliability of the GPAQ (see Table 8). The research design for these studies was similar, using a test–retest procedure to measure the consistency of the GPAQ, which was similar to what has been reported in the literature [4,5]. The sample size ranged from 16 to 940 and there was one study with a sample size smaller than 30. The reason for noting the sample sizes was that Hogg et al. [49] pointed out that small samples (i.e., *n* < 30) for PA measures tended to generate variability that is inappropriate to draw any conclusions on.

The test–retest reliability values varied from moderate to very good and only one study reported poor reliability (*r* < 0.40). For the overall PA, the reliability was good to very good (*r* = 0.58–0.89). Similar findings were found for the overall vigorous PA [VPA] and moderate PA [MPA]. When the overall PA was converted into MET values, however, only moderate reliability was found. The reliability range for work-related PA was about the same for the MPA (*r* = 0.41–0.99) and VPA (*r* = 0.59–0.92). For transport and recreation PA domains, the reliability value ranges were about the same. The time intervals for the short-term reliability ranged from three days to three weeks, and the median value was seven days. Only one study had an interval of three days [36] and three weeks [43], respectively. There were two long-term test–retest reliability studies with a two- (i.e., *r*_-two month apart_ = 0.55) [12] and three-month interval (i.e., *r*_-three month apart_ = 0.53) [42].

## 4. Discussion

The importance of accurately monitoring PA levels on a regular basis in the general population cannot be overstated. The current study contributed to our knowledge base by providing an overview and synthesis concerning the reliability and validity of the GPAQ in different countries. The main potential for this is to save billions of dollars in medical treatments caused by sedentary lifestyles and increase one’s quality of life [5,50]. Overall, the highlighted findings were: (a) Inconsistent reliability and validity among adults in free-living settings found in different countries, and (b) the lack of revalidations in specific age groups as well as in other continents such as Africa, and North and South America. Each of the highlights will be discussed in the following section.

### 4.1. GPAQ Reliability and Validity in Adults in Free-Living Settings

It is deemed critical to note the limitations of the GPAQ to help readers better understand the findings that emerged from the systematic review. First, only self-reported data were used by the GPAQ and some participants reported spending more than 8 h in work-related PA, which might not be true unless they worked over-time. However, it is almost impossible to verify the accuracy of the GPAQ data. Second, MVPA lasted for at least 10 min are measured by the GPAQ. It is possible that the GPAQ may underestimate the total time spent in MVPA and cannot estimate the total PA. Third, work-, transport-, and leisure-related PA can only be accurate when participants can clearly differentiate among the three categories. Fourth, individuals who do not have a job will not be able to report work-related PA. This may be the reason that the GPAQ has only been validated in adult populations, thus limiting its ability to track or monitor individuals’ PA among children and youth. And fifth, the GPAQ uses a typical week to estimate PA data. However, a typical week can be seasonal in many developing countries [9,12], yielding different PA data.

#### 4.1.1. Sample Size

Although there is no single rule of thumb related to adequate sample size for questionnaire validation studies, scholars have recommended an acceptable ratio of survey items and participants to be 1:5 and preferably 1:10 [28]. Therefore, the minimum sample size should be 90–160 and 95–190 for the short and long versions of the GPAQ, respectively. However, about one-third of the revalidation studies (i.e., 8 out of 23) had a sample size smaller than 100 and one study had a sample size smaller than 30 (see Table 5, Table 6 and Table 7). This is a cause for concern as a smaller sample size may negatively affect the representativeness of the population [4,28].

#### 4.1.2. Concurrent Validity

To date, PA wearables are commonly used for testing the concurrent validity of self-reported PA questionnaires [4,5,51]. Relatively low concurrent validity was found when Pearson’s r and/or Spearman’s rho were calculated [34,40] using accelerometers as the criterion standard. This was surprising, given accelerometers are typically found to be more accurate than pedometers [52], and interesting enough, there were no large differences in concurrent validity results that were revealed between studies using the two wearables (see Table 5 and Table 6).

Such results may be explained by the following reasons. First, it might be caused by the differences in measurement methods between the GPAQ and criterion-related measures. The GPAQ is designed to ask participants about their PA and SB lasting for at least 10 min of MVPA excluding light PA in a typical week. Accelerometers and pedometers, on the other hand, were used to measure PA for the entire duration chosen by the researchers in the selected studies. Hence, the week wearing accelerometers/pedometers may not be a typical week because no studies noted that a typical PA week was chosen for participants to wear an accelerometer and/or pedometer or to use a PA log. This means that the GPAQ data were not validated against the same measures obtained from accelerometers/pedometers. Second, the inconsistency in PA measurements between the two methods must have resulted in errors in measuring PA. Accelerometers may underestimate upper body movements and movements involve few vertical motions like cycling, as the accelerometers were usually worn on the dominant hip of the participants [51]. Furthermore, studies have found that pedometers may not accurately record steps for people with abnormal gait patterns and people that are obese [53]. Pedometers also may not accurately capture activities in which the lower body is stationary (i.e., pushing, lifting, and carrying). In fact, low concurrent validity using accelerometers and pedometers as criteria has been found consistently in the literature, resulting from inherent limitations of PA wearables serving as a ‘gold standard’ for self-report questionnaires [15]. Future validation studies should include only bouts of at least 10 min of MVPA from accelerometer data to make it more comparable to the GPAQ measuring activities lasting for at least 10 min.

There is a concern of using pedometer data as the criterion-related standard for testing GPAQ’s concurrent validity in the reported studies (see Table 6). Pedometers measure steps without intensity unless steps are recorded at a specific intensity, such as MPA steps, VPA step, or MVPA steps. Moreover, unlike GPAQ, pedometers measure all steps. As such, steps per day recorded by pedometers are not comparable to the MVPA time measured by GPAQ,

It is also important to point out the use of a previously validated self-reported questionnaire such as the IPAQ as the comparison standard for concurrent validity [9,37,43] as it is not a true gold standard. Many studies have shown the significant differences between data collected from the IPAQ versus accelerometers [51]. In addition, the reason for developing and validating the GPAQ was that the IPAQ-short form (IPAQ-SF) does not measure occupational, transport-related PA, which is the dominant form of PA in many developing countries while the long form of IPAQ was deemed too long and too complex to be used in studies with a large sample size [2,6]. Therefore, it is not surprising that low concurrent validity would be found when IPAQ is used as the so-called gold standard in developing countries. On the other hand, the use of PA logs as a criterion standard for concurrent validity is also a cause for concern as GPAQ and PA log data are not comparable if a typical week of PA is not measured. Unfortunately, none of the selected validation studies on the topic had noted that PA data were measured using the criterion-related standard means during a typical PA week, which is specified in GPAQ (see Table 4).

#### 4.1.3. Reliability

It is encouraging that the range of reliability was found to be good to very good, except for only one study with poor reliability (*r* < 0.30). Of greater importance, the time intervals for the test–retest varied from three days to three weeks for short term-reliability or repeatability, which are within the recommended range [28]. It is alarming, however, that the actual PA change in test–retest was ignored by the two studies [42,43] on long-term repeatability/reliability (i.e., two or three months apart) of the GAPQ without ensuring that participants’ PA pattern remained the same within the two- or three-month time span. This methodological flaw in the research design made the findings dubious [4].

### 4.2. The Lack of Revalidations in Elderly Groups and Other Continents

#### 4.2.1. Revalidation in Various Age Groups

It is surprising that a wide age range (i.e., 15–79) existed in the selected studies and no research has specifically examined the reliability and validity of GPAQ in elderly adults. The following two reasons are for the necessity of validating the GPAQ in elderly groups. First, age affects the accuracy of self-reported PA due to the complexity in estimating PA patterns consisting of intensity, time, frequency, and types [54]. Elderly individuals may not be able to correctly remember their typical PA levels. And second, light/functional PA, which is not measured by GPAQ, plays an important role in the elderly’s overall health [54,55]. This means that the current domain-specific PA (i.e., work, transportation, and recreation) may not be suitable for the elderly considering that they usually do not have a job and only perform limited functional PA.

#### 4.2.2. Revalidations of GPAQ in Other Continents

Although it has been noted that the GPAQ has been used in many African countries [2], it is unclear why it has primarily been validated in Asia and Europe. Hence, this gap in knowledge warrants more attention to professionals in the field of PA measurement and assessment. More validation studies are needed in continents other than Asia and Europe in the future to ensure that the GPAQ is truly a global PA scale.

### 4.3. Limitations

The present review has some limitations that should be acknowledged. Although we thoroughly searched the aforementioned four largest databases in the fields of physical activity measurement and assessment more than once by multiple investigators, it is still possible that not all relevant studies have been identified using the search strategies described in the study. Even though the quality of each study was assessed, they were not weighted or ranked. Thus, findings from studies with poorer quality and smaller sample sizes were given no less importance than other findings. Caution needs to be exercised when interpreting the results of the current project. Factors such as education level, gender, seasons, and types of residential areas (i.e., rural, suburban, and urban) have been found to affect the reliability and validity of GPAQ. Future research is needed on concurrent validity differences in the above factors.

## 5. Conclusions

As a global instrument for measuring PA, the GPAQ has been translated into different languages and validated among adults in more than 20 countries, primarily in Asia and Europe. The GPAQ demonstrated good-to-very good test–retest reliability with time intervals that ranged from three days to two weeks. Poor to fair concurrent validity was found when the GPAQ data were compared to the data measured by PA wearables (i.e., accelerometers and pedometers) and PA logs for seven days. Mixed findings were found concerning the effects of educational level and sex on the reliability and validity of the GPAQ. Incomparable data were used to test the concurrent validity of the GPAQ using the data measured by accelerometers, pedometers, and PA log. As such, it is premature to draw any conclusions concerning the concurrent validity of the GPAQ. Great care must be taken into consideration when interpreting the existing findings of GPAQ concurrent validity. Future research should focus on validating the GPAQ with matching data measured by relatively objective tools such as accelerometers and/or pedometers. More studies are needed to use bouts of at least 10 min of MVPA from accelerometer data to make it more comparable to GPAQ data focusing on MVPA lasting for at least 10 min.

## Figures and Tables

**Table 1:** Search keywords and databases used to select articles.

Initial Search: Assessment Retrieval	Database and Search Terms	Search Criteria
Article’s title	EBSCOhost: Validity, concurrent validity, reliability, validation, global physical activity questionnaire, and Global Physical Activity Questionnaire (GPAQ)	Peer-reviewed journal articles; written in English; analyzed/discussed the reliability and/or validity of the GPAQ; studies using the GPAQ to collect PA data were excluded; conference abstracts and papers were eliminated; articles discussing GPAQ without actual reliability and validity data were not selected; time frame was set from 2002 to September 2019.
	PubMed: Validity, concurrent validity, reliability, validation, global physical activity questionnaire, and GPAQ	
	Google Scholar: Validity, concurrent validity, reliability, validation, global physical activity questionnaire, and GPAQ	
	Webs of Science: Validity, concurrent validity, reliability, validation, global physical activity questionnaire, and GPAQ	

**Table 2:** Descriptions of included studies.

Country	First Author, Year	Research Design		
		Participants	Data Collection	Measures
Bangkok (Thailand)	Sitthipornvorakul et al. 2014 [32]	320 office workers; aged 34.8 ± 6.2 years; 20% male	PA assessed by Yamax Digiwalker CW-700 pedometer for seven days and by GPAQ.	Concurrent validity: ICC for correlation between GPAQ and pedometer data.
Bangladesh	Mumu et al. 2017 [34]	162 healthy adults; aged 35 ± 9 years; 54% female	Seven-day wearing AG, then answered GPAQ in a face-to-face interview.	Concurrent validity: Spearman’s rho between GPAQ and accelerometer indicators.
Chile	Aguilar-Farias et al. 2017 [19]	217 adults; aged 43.77 ± 15.75 year; 42.9% male	Seven-day wearing AG; face-to-face interview GPAQ single question about sedentary behavior.	Concurrent validity (Spearman correlation) between AG and GPAQ.
China	Hu et al. 2015 [11]	205 adults; aged 30–70 years; 38.54% male	Completed three questionnaires twice (Day 1 and Day 9), a PA-log for seven days.	Test–retest reliabilities: Using intra-class correlation coefficients (ICC); Relative validities: Comparing the data from PA questionnaires and PA-log.
France	Rivière et al. 2018 [35]	92 adults (56.5% students; 43,5% staff in a medical school); age >18; 27.2% male	Seven-day wearing AG, complete GPAQ before and after wearing AG.	Reliability and criterion and concurrent validity of GPAQ against AG.
India	Misra et al. 2014 [36]	234 participants; age 15–74 years; 49.6% male	Test–retest repeatability of GPAQ, IPAQ, and pedometer.	Spearman’s rho, ICC for validity and reliability.
	Mathews et al. 2016 [45]	47 adults; aged 18–64 years; 100% female	Using AG to validate the self-polished modified GPAQ.	Concurrent validity (Spearman’s rho) and ICC
Korea	Lee et al. 2019 [46]	115 for reliability (55 males and 60 females), age 19–75 years; 199 adults for validity (82 males and 117 females)	Completed GPARQ twice with seven days apart; Seven-day wearing AG, complete GPAQ after wearing AG.	Test–retest reliability and criterion-related validity (Spearman’s rho)
Malaysia	Lingesh et al. 2016 [9]	43 nurses; aged 24 to 55 years (44.48 ± 8.38 years); 100% female	IPAQ and GPAQ: Measured on the eighth day, and wore SenseWear accelerometer and recorded PA logs for seven consecutive days.	PA data measured by PA logs for seven days; METs-min/week^−1^ was used; Pearson correlations and a Bland–Altman plot.
	Soo et al. 2015 [37]	100 adults; aged 20–58 years; 83% female	By comparing GPAQ-M with IPAQ-S and objectively measuring PA using a Yamax DigiWalker pedometer.	Two-week test–retest reliability: Using the Wilcoxon signed-rank analysis; concurrent validity: Spearman’s rho by comparing GPAQ-M data with IPAQ and objectively measured PA data.
Saudi Arabia	Alkahtani, 2016 [38]	62 college students; aged 19–21 years (20.0 ± 1.1 year); 100% male	Completed GPAQ twice (two weeks apart) and wore AG for seven consecutive days.	Test–retest reliability and concurrent validity of the GPAQ with AG using Spearman’s rho.
Singapore	Chu et al. 2015 [18]	110 working adults and students; aged 31 (26.8–47.3); 70.9% female	Self- and interviewer-administration of GPAQ, seven days of AG.	Test–retest reliability with one-week interval; criterion validity with Spearman’s ICC.
	Chu et al. 2018 [7]	84 medicine faculty and staff at a university; aged 21–65 years; 69% female	Single sitting item of GPAQ using self- and interviewer-administered modes twice with seven days apart, seven days of AG.	Reliability using the Spearman’s rho and ICC; Convergent validity using Spearman’s rho.
South Africa	Watson et al. 2017 [39]	95 pregnant women, aged 29.5 ± 5.7 years	Data collected at 14–18 weeks and 29–33 weeks’ gestation; seven-day wearing AG; comparing total time in MVPA between GPAQ and AG.	Content validity, convergent validity in comparison with AG; relative validity.
Spain	Ruiz-Casado et al. 2016 [40]	204 cancer survivors; aged 18–79 years; 36% male	Comparing IPAQ-SF and GPAQ with AG; AG was worn for 5 to 10 days.	Validity: Wilcoxon signed-rank was used to compare the differences between questionnaire and accelerometry data.
Switzerland	Wanner et al., 2017 [47]	354 (physical activity) and 366 (sitting), age 18–83 years	Completed GPAQ on Day 1, then wore AG for seven days.	Concurrent validity (Spearman correlation)
The United Arab Emirates (UAE)	Doyle et al. 2019 [48]	93 university students;	Completing GPAQ-A on two occasions (seven days apart); wearing an accelerometer for seven days.	Test–retest reliability and criterion validity
UK	Cleland et al. 2014 [16]	101 adults; aged 44 ± 14 years; 54% male	Wore AG for seven days and completed GPAQ on Day 7; Repeated for a random sub-sample at three to six months later.	Wilcoxon-signed rank tests for differences in measures; Spearman’s rho coefficient for criterion validity and extent of change.
US	Gorzelitz et al. 2018 [41]	347 adults; aged 50.7 ± 16.9 years; 46.7% male	Wore AG for seven days; GPAQ face-to-face interviews self-reported data.	MVPA data measured by both GPAQ and AG; MVPA converted into METs.
	Herrmann et al. 2013 [42]	Study 1: 69 adults; aged 43.1 ± 11.4 years; 82.6% female; Study 2: 16 adults; aged 40.2 ± 12.6 years; 50% female	First study for long-term test–retest reliability with three moths apart, completed GPAQ and worn ActiGraph GT1M accelerometer for seven days; Second study for short-term test–retest reliability with 10 days apart.	ICC for reliability; weighted Cohen’s K and percent agreement for testing validity with categorical scores (IPAQ vs. GPAQ); Spearman’s rho for validity with numerical variables.
	Hoos et al. 2012 [43]	72 Latinas; aged 43.01 ± 9.05 years; 58% female	Worn accelerometer for seven days at the baseline and six months later; GPAQ data collected at the same time.	GPAQ’s sensitivity to intervention change using Spearman’s rho for concurrent validity.
	Metcalf et al. 2018 [20]	108 residents; aged 49.4 years (range: 19.8–68.7); 68.5% female	Seven-day wearing AG followed by a telephone interview of GPAQ.	Multivariable linear regression models using functions of the GPAQ data to predict AG measured physical activity and sedentary behavior.
Vietnam	Thuy et al. 2010 [22]	251 adults; aged 25–64 years; 50.6% female	GPAQ and IPAQ were administered face-to-face, then wore a pedometer and complete PA log for seven consecutive days.	Reliability of GPAQ and IPAQ for groups; Concurrent validity was assessed from the correlations between pedometer steps per day and IPAQ.
	Trinh et al. 2009 [12]	169 adults; aged 25–64 years; 48.5% male	GPAQ was administered twice in the dry and wet season two weeks apart, respectively; wore the accelerometer twice for seven days during the week before the first and last GPAQ administration.	Spearman’s rho for the repeatability of the GPAQ, weighted Cohen’s Kappa for reliability; Spearman’s rho for the criterion validity of the GPAQ.
Bangladesh, Brazil, China, Ethiopia, India, Indonesia, Japan, Portugal, and South Africa	Bull et al. 2009 [1]	2657 adults from nine countries; aged 18–75 years; 61.3% male	Ten projects were initiated in 2002–2003 through WHO headquarters and regional offices and other known networks.	Test–retest reliability of GPAQ for categorical variables using Cohen’s Kappa and Spearman’s rho for continuous variables; Concurrent validity with IPAQ and pedometer/accelerometer data using Spearman’s rho.
Belgium, Spain, UK	Laeremans et al. 2017 [44]	122 adults; aged 35 ± 10 years; 45% males	Seven-day wearing SenseWear armband and complete GPAQ online on the final day; adjusted GPAQ to capture information on walking, cycling and e-biking trips separately for the travel to and from work subscale; three trials for the same data collection.	Reliability: The changes in the difference between two methods over three trials; energy expenditure and minutes spent in MVPA, MPA, VPA and sedentary behaviors; Validity: Wilcoxon signed rank-sum test, Spearman correlation coefficients, mixed-effects regression models and Bland–Altman plots.

Note: AG = accelerometer Actigraph GT3X; GPAQ = Global Physical Activity Questionnaire; GPAQ-A = Global Physical Activity Questionnaire-Arabic Version; ICC = intraclass correlation coefficient; IPAQ-SF = International Physical Activity Questionnaire-Short Form; LoA = limits of agreement; MVPA = moderate-to-vigorous physical activity; MPA = moderate physical activity; VPA = vigorous physical activity.

**Table 3:** Means of methodological quality assessment.

Assessment Questions	Article by Author						
	**Aguilar-Farias et al. 2017 [19]**	**Alkahtani 2016 [38]**	**Bull et al. 2009 [1]**	**Chu et al. 2015 [18]**	**Chu et al. 2018 [7]**	**Cleland et al. 2014 [16]**	**Doyle et al. 2019 [48]**
1. Was the research question or objective in this paper clearly stated?	3	3	3	3	3	3	3
2. Was the study population clearly specified and defined?	3	3	2	2	3	2	2
3. Was the participation rate of eligible persons at least 50%?	3	3	3	3	3	3	2
4. Were all the subjects selected or recruited from the same or similar populations (including the same time period)? Were inclusion and exclusion criteria for being in the study prespecified and applied uniformly to all participants?	2	2	1	2	3	2	2
5. Was a sample size justification, power description, or variance and effect estimates provided?	1	3	1	3	3	1	1
6. For the analyses in this paper, was the exposure(s) of interest measured prior to the outcome(s) being measured?	3	3	3	3	3	3	3
7. Was the timeframe sufficient so that one could reasonably expect to see an association between exposure and outcome if it existed?	3	3	3	3	3	3	2
8. For exposures that can vary in amount or level, did the study examine different levels of the exposure as related to the outcome (e.g., categories of exposure, or exposure measured as a continuous variable)?	3	3	3	3	3	3	3
9. Were the exposure measures (independent variables) clearly defined, valid, reliable, and implemented consistently across all study participants?	3	3	3	3	3	2.5	2.5
10. Was the exposure(s) assessed more than once over time?	1	3	3	2.5	3	3	2
11. Were the outcome measures (dependent variables) clearly defined, valid, reliable, and implemented consistently across all study participants?	2.5	3	3	3	3	3	3
12. Were the outcome assessors blinded to the exposure status of participants?	3	3	3	3	3	3	3
13. Were key potential confounding variables measured and adjusted statistically for their impact on the relationship between exposure(s) and outcome(s)?	2.5	3	3	3	3	2	1.5
Average score	2.5	2.9	2.6	2.8	3	2.6	2.3
	**Gorzelitz et al. 2018 [41]**	**Herrmann et al. 2013 [42]**	**Hoos et al. 2012 [43]**	**Hu et al. 2015 [11]**	**Lee et al. 2019 [46]**	**Lingesh et al. 2016 [9]**	**Laeremans et al. 2017 [44]**
1. Was the research question or objective in this paper clearly stated?	3	3	3	3	3	3	3
2. Was the study population clearly specified and defined?	3	2	3	3	2	3	3
3. Was the participation rate of eligible persons at least 50%?	3	1	3	3	3	3	3
4. Were all the subjects selected or recruited from the same or similar populations (including the same time period)? Were inclusion and exclusion criteria for being in the study prespecified and applied uniformly to all participants?	3	2	3	3	2	2	2
5. Was a sample size justification, power description, or variance and effect estimates provided?	2	1	3	1	1	1.5	1
6. For the analyses in this paper, was the exposure(s) of interest measured prior to the outcome(s) being measured?	3	2	3	3	3	3	3
7. Was the timeframe sufficient so that one could reasonably expect to see an association between exposure and outcome if it existed?	3	3	3	3	3	3	3
8. For exposures that can vary in amount or level, did the study examine different levels of the exposure as related to the outcome (e.g., categories of exposure, or exposure measured as a continuous variable)?	3	2	3	3	3	2	3
9. Were the exposure measures (independent variables) clearly defined, valid, reliable, and implemented consistently across all study participants?	3	3	3	3	3	2	3
10. Was the exposure(s) assessed more than once over time?	3	1.5	3	3	3	1	3
11. Were the outcome measures (dependent variables) clearly defined, valid, reliable, and implemented consistently across all study participants?	3	3	3	3	3	2	3
12. Were the outcome assessors blinded to the exposure status of participants?	3	3	3	3	3	3	3
13. Were key potential confounding variables measured and adjusted statistically for their impact on the relationship between exposure(s) and outcome(s)?	3	1.5	3	3	3	3	3
Average score	2.8	2.2	3	2.8	2.7	2.4	2.8
	**Mathews et al. 2016 [45]**	**Metcalf et al. 2018 [20]**	**Misra et al. 2014 [36]**	**Mumu et al. 2017 [34]**	**Rivière et al. 2018 [35]**	**Ruiz-Casado et al. 2016 [40]**	**Sitthipornvorakul et al. 2014 [32]**
1. Was the research question or objective in this paper clearly stated?	3	3	3	3	3	3	3
2. Was the study population clearly specified and defined?	2	3	1	3	3	3	2
3. Was the participation rate of eligible persons at least 50%?	2	3	3	3	3	3	1
4. Were all the subjects selected or recruited from the same or similar populations (including the same time period)? Were inclusion and exclusion criteria for being in the study prespecified and applied uniformly to all participants?	1	3	1	2	2	3	3
5. Was a sample size justification, power description, or variance and effect estimates provided?	1	1	1	1	1	1	1
6. For the analyses in this paper, was the exposure(s) of interest measured prior to the outcome(s) being measured?	1	3	3	3	3	3	3
7. Was the timeframe sufficient so that one could reasonably expect to see an association between exposure and outcome if it existed?	2	3	3	3	3	1.5	1
8. For exposures that can vary in amount or level, did the study examine different levels of the exposure as related to the outcome (e.g., categories of exposure, or exposure measured as a continuous variable)?	2	3	2	3	3	3	1.5
9. Were the exposure measures (independent variables) clearly defined, valid, reliable, and implemented consistently across all study participants?	2	3	3	3	3	3	2
10. Was the exposure(s) assessed more than once over time?	1	3	3	1	3	1	2
11. Were the outcome measures (dependent variables) clearly defined, valid, reliable, and implemented consistently across all study participants?	2.5	3	2.5	3	3	3	3
12. Were the outcome assessors blinded to the exposure status of participants?	2	3	3	3	3	3	3
13. Were key potential confounding variables measured and adjusted statistically for their impact on the relationship between exposure(s) and outcome(s)?	2	3	3	3	3	3	1
Average score	1.8	2.8	2.4	2.6	2.8	2.6	2.0
	**Soo et al. 2015 [37]**	**Thuy et al. 2010 [22]**	**Trinh et al. 2009 [12]**	**Wanner et al. 2017 [47]**	**Watson et al. 2017 [39]**	-	-
1. Was the research question or objective in this paper clearly stated?	3	3	3	3	3		
2. Was the study population clearly specified and defined?	3	2	3	3	3		
3. Was the participation rate of eligible persons at least 50%?	3	3	3	3	3		
4. Were all the subjects selected or recruited from the same or similar populations (including the same time period)? Were inclusion and exclusion criteria for being in the study prespecified and applied uniformly to all participants?	3	2	2	3	3		
5. Was a sample size justification, power description, or variance and effect estimates provided?	2	1	1	2	2.5		
6. For the analyses in this paper, was the exposure(s) of interest measured prior to the outcome(s) being measured?	3	3	3	3	3		
7. Was the timeframe sufficient so that one could reasonably expect to see an association between exposure and outcome if it existed?	3	3	3	3	3		
8. For exposures that can vary in amount or level, did the study examine different levels of the exposure as related to the outcome (e.g., categories of exposure, or exposure measured as a continuous variable)?	3	3	3	3	3		
9. Were the exposure measures (independent variables) clearly defined, valid, reliable, and implemented consistently across all study participants?	3	2	3	3	3		
10. Was the exposure(s) assessed more than once over time?	3	3	3	3	3		
11. Were the outcome measures (dependent variables) clearly defined, valid, reliable, and implemented consistently across all study participants?	3	2.5	3	3	2		
12. Were the outcome assessors blinded to the exposure status of participants?	3	3	3	3	3		
13. Were key potential confounding variables measured and adjusted statistically for their impact on the relationship between exposure(s) and outcome(s)?	3	3	3	2	3		
Average score	2.9	2.6	2.8	2.8	2.8		

Note: Score scale: Strong = 3, Good = 2, Weak = 1.

**Table 4:** Results of methodological weaknesses.

Country	Studies	Methodological Weaknesses
Bangkok (Thailand)	Sitthipornvorakul et al. 2014 [32]	The Yamax Digiwalker CW-700 pedometer was removed when immersing the body in water; participants who had four instead of seven daily measurements were also included in the study; PA intensities were classified using pedometer steps; there is a lack of information on whether the pedometer data were collected during a typical week when GPAQ data were measured.
Bangladesh	Mumu et al. 2017 [34]	Water-based activities were excluded, resulting in underestimates of PA by the accelerometer; participants who wore the accelerometer for ≥3 days were also included; there is a lack of information on whether the accelerometer data were collected during a typical week when GPAQ data were measured.
Chile	Aguilar-Farias et al. 2017 [19]	The accelerometer data were not measured during a typical week when GPAQ data were measured.
China	Hu et al. 2015 [11]	Self-reported PA log data were used as the criterion-referenced standards for GPAQ data; there is a lack of information on whether the accelerometer data were collected during a typical week when GPAQ data were measured.
France	Rivière et al. 2018 [35]	Less than 100 participants were recruited.
India	Misra et al. 2014 [36]	Pedometers was used as the criterion-referenced standard.
	Mathews et al. 2016 [45]	Less than 100 participants were recruited (*n* = 47 women); total PA was not measured; there is a lack of information on whether the accelerometer data were collected during a typical week when GPAQ data were measured
Korea	Lee et al. 2019 [46]	There is a lack of information on whether the accelerometer data were collected during a typical week when GPAQ data were measured
Malaysia	Lingesh et al. 2016 [9]	Less than 100 participants were recruited (*n* = 43 females only); there is a lack of information on whether the accelerometer data were collected during a typical week when GPAQ data were measured.
	Soo et al. 2015 [37]	Pedometers were used as the criterion-referenced standard; average pedometer steps were compared to GPAQ min. data; there is a lack of information on whether the pedometer data were collected during a typical week when GPAQ data were measured.
Saudi Arabia	Alkahtani, 2016 [38]	Only 62 male participants were recruited; those who wore an accelerometer for ≥4 days were included; there is a lack of information on whether the accelerometer data were collected during a typical week when GPAQ data were measured.
Singapore	Chu et al. 2015 [10]	There is a lack of information on whether the accelerometer data were collected during a typical week when GPAQ data were measured.
	Chu et al. 2018 [7]	Only 78 participants were involved in the study with 69.0% of females; there is a lack of information on whether the accelerometer data were collected during a typical week when GPAQ data were measured.
South Africa	Watson et al. 2017 [39]	95 pregnant women were recruited; there is a lack of information on whether the accelerometer data were collected during a typical week when GPAQ data were measured.
Spain	Ruiz-Casado et al. 2016 [40]	There is a lack of information on whether the accelerometer data were collected during a typical week when GPAQ data were measured.
Switzerland	Wanner et al. 2017 [47]	There is a lack of information on whether the accelerometer data were collected during a typical week when GPAQ data were measured.
UAE	Doyle et al. 2019 [48]	Less than 100 participants were recruited (*n* = 93 for reliability study, *n* = 43 for concurrent validity study); there is a lack of information on whether the accelerometer data were collected during a typical week when GPAQ data were measured.
UK	Cleland et al. 2014 [16]	There is a lack of information on whether the accelerometer data were collected during a typical week when GPAQ data were measured.
US	Gorzelitz et al. 2018 [41]	There is a lack of information on whether the accelerometer data were collected during a typical week when GPAQ data were measured.
	Herrmann et al. 2013 [42]	Only 68 participants were included; there is a lack of information on whether the accelerometer data were collected during a typical week when GPAQ data were measured.
	Hoos et al. 2012 [43]	Less than 100 participants (*n* = 72) were included; there is a lack of information on whether the accelerometer data were collected during a typical week when GPAQ data were measured.
	Metclif et al. 2018 [20]	There is a lack of information on whether the accelerometer data were collected during a typical week when GPAQ data were measured.
Vietnam	Thuy et al. 2010 [22]	Accelerometer data were not collected during a typical week.
	Trinh et al. 2009 [12]	Accelerometer data were not collected during a typical week.
Bangladesh, Brazil, China, Ethiopia, India, Indonesia, Japan, Portugal, and South Africa	Bull et al. 2009 [1]	Accelerometer or pedometer data were not collected during a typical week.
Belgium, Spain, UK	Laeremans et al. 2017 [44]	Accelerometer data were not collected during a typical week.

**Table 5:** Summary of concurrent validity of GPAQ with accelerometers in various countries.

Country (1st Author, Year)	Sample Size	GPAQ Measures						
		Sitting	MPA	VPA	MVPA	Work	Transport	Leisure
Bangladesh (Mumu et al. 2017 [34])	162 healthy adults, age = 35 ± 69 years	*r* = 0.23 **			*r* = 0.18 *, 0.24 * (CPM), 0.28 ** (step)			
	65 from urban	*r* = 0.07			*r*= 0.46 **, 0.55 ** (CPM), 0.63 ** (step)	*r* = 0.38 **, 0.50 ** (CPM), 0.55 ** (step)	*r* = 0.49 **, 0.46 ** (CPM), 0.52 ** (step)	*r* = 0.26 *, 0.29 * (CPM), 0.41 ** (step)
	97 from rural areas	*r* = 0.38 **			*r* = 0.0001, −0.01 (CPM), 0.05 (step)	*r* = −0.03, 0.02 (CPM), 0.07 (step)	*r* = −0.20 *, −0.23 * (CPM), −0.13 (step)	*r* = 0.02, −0.05 (CPM), −0.12 (step)
	70 men				*r* = −0.10, 0.04 (CPM), 0.05 (step)			
	85 women				*r* = 0.42 **, 0.46 ** (CPM), 0.49 ** (step)			
	93 (≤35 years)				*r* = 0.31 *, 0.32 ** (CPM), 0.34 ** (step)			
	62 (>35 years)				*r* = −0.03, 0.10 (CPM), 0.19 (step)			
	30 illiterates				*r* = 0.27, 0.35 (CPM), 0.22 (step)			
	37 primary school				*r* = 0.23, 0.20 (CPM), 0.38 * (step)			
	61 high school				*r* = −0.01, 0.01 (CPM), 05 (step)			
Chile (Aguilar-Farias et al. 2017 [19])	217, age = 43.77 ± 15.75 years	*r* = 0.23 ***(1-min), 0.26 *** (5-min), 0.26 *** (10-min); LoA = −768.9 to 181.2 (1-min), −200.4 to 137.1 (5-min), −539.5 to 387.3 (10-min); Poor in classifying sedentary behavior into tertiels for 1-, 5- and 10-min bouts: Agreement = 43.5%, 46.0%, 42.2%; *k* = 0.18, 0.19, 0.13; and quartiles: Agreement = 31.3%, 32.7%, 31.3%; *k* = 0.08, 0.10, 0.08						
	≥ 45 and < 45 years (*n*: N/A)	*r* = 0.38 *** (≥45); 0.10 (<45)						
	93 men and 124 women	*r* = 0.23 * (men); 0.21 * (women)						
	66 (≥12 years of education) and 151(<12 years)	*r* = 0.13 (≥12 years); 0.27 * (<12 years)						
	Mostly standing work and balanced standing and sitting work (*n* not provided)	*r* = 0.26 *** (balanced standing and sitting); −0.02 (mostly standing)						
France (Rivière et al. 2018 [35])	92 students and staff in a medical school, age >18 years	*r* = 0.42 **; LoA = 90.1 to 412.3 min/week	*r* = 0.10	*r* = 0.38 **; LoA = 286.5 to 601.3 min/week				
Korea (Lee et al. 2019 [46])	199 adults, 82 males and 117 females	*r* = 0.18 **	*r* = 0.33 **	*r* = 0.10	*r* = 0.34 **			
	170 adults age 19–64	*r* = 0.19 *	*r* = 0.37 **	*r* = 0.09	*r* = 0.36 **			
	29 elders age >64	*r* = 0.08	*r* = 0.33	*r* = −0.02 *	*r* = 0.38 *			
India (Misra et al. 2014 [36])	116 males and 118 females; age 15–65 years;	*r* = 0.29 **			*r* = 0.36 * (step); *r* = 0.31 * (male, step), 0.40 * (female, step)			
India (Mathews et al. 2016 [45])	47 women, age 18–64 years				*r* = 0.69 (non-bouted), 0.60 (10-min bouts); ICC = 0.78 (non-bouted), 0.55 (10-min bouts)			
Malaysia (Lingesh et al. 2016 [9])	43 female nurses aged 24 to 55 years (mean: 44.48 ± 8.38)				*r* = −0.11			
Saudi Arabia (Alkahtani, 2016 [38])	62 male college students, aged 19–21 years old	*r* = 0.08	*r* = 0.24	*r* = 0.32 **	*r* = 0.32 *			
Singapore (Chu et al. 2015 [10])	110 working adults and students		*r* = 0.36 *** (total), 0.20 (10-min bouts); LoA = −115.0 to 121.0 (total); −88.7 to 148.1 (10-min bouts)	*r* = 0.45 *** (total), 0.39 *** (10-min bouts); LoA = −46.2 to 99.9 (total), 46.5 to 102.1 (10-min bouts) (10-min bouts)	*r* = 0.39 *** (total), 0.37 *** (10-min bouts); LoA = −138.7 to 210.4 (total), −84.8 to 199.8 (10-min bouts)			
	52 self-administrated		*r* = 0.28 * (total), 0.29 * (10-min bouts)	*r* = 0.35 * (total), 0.38 ** (10-min bouts)	*r* = 0.32 * (total), 0.30 * (10-min bouts)			
	56 interview-administrated		*r* = 0.44 *** (total), 0.42 *** (10-min bouts)	*r* = 0.43 *** (total), 0.52 *** (10-min bouts)	*r* = 0.44 *** (total), 0.46 *** (10-min bouts)			
Singapore (Chu et al. 2018 [7])	84 medicine faculty and staff, aged 21–65 years	*r* = 0.28 *		84 medicine faculty and staff, aged 21–65 years				
	37 self-administrated and 41 interview-administrated	*r* = 0.46 * (self), 0.12 (interview)						
South Africa (Watson et al. 2017 [39])	95 pregnant women at 14–18 and 29–33 weeks’ gestation, age = 29.5 ± 5.7 years	ICC = 0.08 (14–18 weeks), 0.01 (29–33 weeks); poor agreement in categorizing active/inactive participants, *k* = 0.11 (14–18 weeks), −0.02 (29–33 weeks)			ICC = 0.05 (14–18 weeks), −0.05 (29–33 weeks); poor agreement in classifying PA to quartiles, *k* = 0.09 (14–18 weeks), −0.03 (29–33 weeks)			
Spain (Ruiz-Casado et al. 2016 [40])	204 cancer survivors aged 18–79 years	*r* = 0.17 *; LoA = −4400 to 425	*r* = −0.03; LoA = −911 to 1395	*r* = 0.73 ***; LoA = −60 to 75				
UAE (Doyle et al. 2019 [48])	43 Arabic speaking university students	*r* = −0.02			*r* = *0.23*			
UK (Cleland et al. 2014 [16])	95 participants, age = 44 ± 14 years; 44 females, 51 males	*r* = 0.187 ***; low extent of change over 3–6 months, *r* = −0.024 *; *r* = 0.378 * (women), −0.053 (men)			*r* = 0.484 ***; moderate extent of change over 3–6 months, *r* = 0.52 *; *r* = 0.434 * (women), 0.496 ** (men)			
Switzerland (Wanner et al., 2017 [47])	354 (physical activity) and 366 (sitting), age 18–83 years	*r* = 0.47 ***	*r* = 0.16 **	*r* = 0.46 ***	*r* = 0.22 (CPM) ***, 0.25 *** (steps); 0.11 * (min/week)	*r* = −0.13 *	*r* = 0.15 **	
USA (Gorzelitz et al. 2018 [41])	347 (age >18), 162 (46.7%) male				MVPA Discordance between GAPQ and accelerometer data varied by sex, education level and marital status			
USA (Herrmann et al. 2013 [42])	54, age = 43.1 ± 11.4 years)	*r* = 0.12	*r* = 0.36 **	*r* = 0.39 **	*r* = 0.26 *			
USA (Hoos et al. 2012 [43]) Vietnam (Trinh et al. 2009 [12])	72 Latinas, aged 18–65 years (mean = 43.01 ± 9.05 years)	*r* = 0.28 (pre), 0.25 (post)	*r* = 0.04 (pre), 0.04 (post)	*r* = 0.42 ** (pre), 0.24 (post)	*r* = 0.14 (pre), −0.06 (post)	*r* = −0.17 (pre, MVPA), −0.15 (pre, MPA), 0.03 (pre, VPA): *r* = −0.21 (post MVPA), −0.10 (post, MPA), −0.19 (post, VPA)	*r* = 0.24 ** (pre, MPA); *r* = 0.04 (post, MPA)	
	169 aged 25–64 years (44.7 ± 11.1 years)	*r* = 0.23 (dry season), 0.32(wet season)	*r* = 0.18 (dry), 0.10 (wet)	*r* = −0.04 (dry), 0.03 (wet)	*r* = 0.20 (dry, MVPA), 0.09 (wet, MVPA); *r* = 0.34 (dry, total counts), 0.20 (wet, total counts)			
Belgium, Spain, UK (Laeremans et al. 2017 [44])	122 adults (41 Belgium, 41 Spain, 40 UK); 45% males, age: 35 ± 10 years	*r* = 0.12 (0.09–0.24 **)	*r* = 0.33 (0.11−0.34 **)	*r* = 0.64 (0.59 ***–0.69 **)	*r* = 0.65 (0.55 ***–0.64 ***)			

Note: * *p* < 0.05; ** *p* < 0.01; *** *p* < 0.001; CPM = counts per minute; ICC = intraclass correlation coefficient; IPAQ-SF = International Physical Activity Questionnaire; LoA = limits of agreement; MVPA = moderate-to-vigorous physical activity; MPA = moderate physical activity; VPA = vigorous physical activity.

**Table 6:** Summary of concurrent validity of MVPA measured by GPAQ with pedometers in various countries.

Country (1st Author, Year)	Sample Size	GPAQ Measures		
		Sitting Time	Steps/Day	MVPA
Bangkok (Sitthipornvorakul et al. 2014 [32])	320 office workers			*r* = 0.08
	By age: 77 (20–29 years), 115 (30–39 years), and 88 (over 40 years)			*r* = 0.27 * (20–29 years), −0.01 (30–39 years), 0.09 (40+ years) ^a^
Malaysia (Soo et al. 2015 [37])	100 aged 20–58 years		*r* = 0.265 *	
Vietnam (Thuy et al. 2010 [22])	120 men and 118 women; by work pattern: 146 with stable and 92 with unstable work patterns;		Men with table job: *r* = 0.42, unstable job: *r* = 0.22; Women with stable job: *r* = *0.33,* unstable job: *r* = 0.16	
Bangladesh, Brazil, China, Ethiopia, India, Indonesia, Japan, Portugal, and South Africa (Bull et al. 2009 [1])	2657 male and female adults from 9 countries; *n* = 1951 for criterion validity	*r* = −0.20 **, ranging from 0 (Japan) to −0.37 (Taiwan, China)	*r* = 0.31 ** (excluding China, Brazil, Portugal, South Africa), ranging from 0.06 (Bangladesh) to 0.35 (India)	
	980 males and 971 females; 1077 with fewer than 13-year education and 298 with more than 13-year education		Similar criterion validity between genders and between low and high education level.	
	976 from urban areas and 819 from rural areas		*r* = 0.23(urban), 0.43(rural)	
	406 underweight, 932 healthy weight, and 262 overweight/obese		*r* = 0.52 (underweight), 0.34 (healthy BMI), 0.08 (overweight/obese)	

Note: * *p* < 0.05; MVPA = moderate and vigorous physical activity; ^a^ = PA intensity was classified by the total daily steps. BMI = body mass index.

**Table 7:** Summary of concurrent validity of GPAQ with PA log and IPAQ in various countries.

Country (1st Author, Year)	Sample Size	Criterion Measures	GPAQ Measures			
			Sitting	MPA	VPA	MVPA
China (Hu et al. 2015 [11])	205 aged 30–70 years, 38.54% of males	PA log	*r* = 0.52 **	*r* = 0.47 **; 0.43 ** (MPA excluding walking)	*r* = 0.41 **	*r* = 0.51 **
India (Misra et al. 2014 [36])	262 aged 15–65 years, 116 males (49.6%)	IPAQ	*r* = 0.999 ***	*r* = 0.894 ***	*r* = 0.934 ***	*r* = 0.939 ***
Malaysia (Lingesh et al. 2016 [9])	43 female nurses aged 24 to 55 years (44.48 ± 8.38 years)	PA Log				*r* = −0.015
		IPAQ				*r* = 0.214
Malaysia (Soo et al. 2015 [37])	100 aged 20–58 years	IPAQ	*r* = 0.447 ***	*r* = 0.459 ***	*r* = 0.466 ***	*r* = 0.309 **
Vietnam (Thuy et al. 2010 [22])	251 (120 men and 118 women)	PA Log				*r* = 0.49 (men), −0.05 (women)
		IPAQ				*r* = 0.39 (men), 0.18 (women)
	By work pattern: 146 with stable and 92 with unstable work patterns	PA Log				*r* = 0.31 (stable)
		IPAQ				*r* = 0.32 (stable)
Bangladesh, Brazil, China, Ethiopia, India, Indonesia, Japan, Portugal, and South Africa (Bull et al. 2009 [1])	2657 male and female adults from nine countries; *n* = 1951 for criterion validity	IPAQ	*r* = 0.65 **; poor agreement in categorizing inactive time, agreement = 85.2%, *k* = 0.22	*r* = 0.45 **	*r* = 0.57 **	*r* = 0.54 **

Note: ** *p* < 0.01; *** *p* < 0.001; MVPA = moderate-to-vigorous physical activity; MPA = moderate physical activity; VPA = vigorous physical activity.

**Table 8:** Summary of GPAQ test–retest reliability in different countries.

Country (Author, Year)	Sample Size	Days Apart		Reliability Results					
				Overall PA	MET	Work	Transport	Recreation	Sitting
China (Hu et al. 2015 [11])	205 participants, 38.54% of males, aged 30–70 years	9		*r* = 0.81					*r* = 0.80
France (Rivière et al. 2018 [35])	92 students and staff in a medical school, age >18	7		*r* = 0.58			*r* = 0.67	Vigorous: *r* = 0.94; moderate: *r* = 0.37	*r* = 0.80
India (Misra et al. 2014 [36])	262 subjects, 116 (49.6% male), age 15–65 years	3		*r* = 0.67	*r* = 0.68	Vigorous: *r* = 0.81; moderate: *r* = 0.37	*r* = 0.72	*r* = 0.43	
Korea (Lee et al. 2019 [46])	115 adults, aged 19–65 years, 48% male	7		*r* = 0.47		*r* = 0.27–0.47		*r* = 0.53 −0.70	*r* = 0.65
Malaysia (Soo et al. 2015 [37])	100 adults aged 20–58 years old	14			z = −0.450, *p* = 0.653	Vigorous: z = −0.093, *p* = 0.926; moderate: z = −0.733, *p* = 0.464		Vigorous-intensity: z = 0.445, *p* = 0.656; moderate- intensity: z = −3.515, *p* < 0.001	z = −3.272, *p* = 0.001
Saudi Arabia (Alkahtani, 2016 [38])	62 male college students, aged 19–21 years old	14		Vigorous: *r* = 0.78; Moderate: *r* = 0.44					*r* = 0.70
Singapore (Chu et al. 2015 [18])	110 working adults and students	7		MVPA: *r* = 0.54		Vigorous: *r* = 0.59; Moderate: *r* = 0.37	*r* = 0.47	Vigorous: *r* = 0.73; moderate: *r* = 0.60	
Singapore (Chu et al. 2018 [7])	84 medicine faculty and staff at a university; aged 21–65 years	7		Self-administered group	MVPA: *r* = 0.63		Moderate: *r* = 0. 55; vigorous: *r* = 0.71	*r* = 0.47	Moderate: *r* = 0.46; vigorous: *r* = 0.86
				Interview-administered group	MVPA: *r* = 0.61		Moderate: *r* = 0.41	*r* = 0.73	Moderate: *r* = 0.59; vigorous: *r* = 0.82
				All	MVPA: *r* = 0.63		Moderate: *r* = 0.48; vigorous: *r* = 0.71	*r* = 0.60	Moderate: *r* = 0.53; vigorous: *r* = 0.83
UAE (Doyle et al. 2019 [48])	227 Arabic speaking university students, aged 18–32, 59.1% women	7		Moderate to vigorous: *r* = 0.78; Moderate: *r* = 0.73; Vigorous: *r* = 0.62					*r* = 0.44
US (Herrmann et al. 2013 [42])	Study 1: 69 and 54 adults three months apart;	Short term (10)		*r* = 0.89		Moderate: *r* = 0.87	*r* = 0.83	Moderate: *r* = 0.96; vigorous: *r* = 0.90	*r* = 0.92
	Study 2: 16 adults; aged 18–65 years	Long terms (3 months)		*r* = 0.82		Moderate: *r* = 0.68; vigorous: *r* = 0.74	*r* = 0.54	Moderate: *r* = 0.53; vigorous: *r* = 0.74	*r* = 0.83
Vietnam (Thuy et al. 2010 [22])	randomly selected 251 adults	21	Male	*r* = 0.32			*r* = 0.28	*r* = 0.18	*r* = 0.20
			Female	*r* = 0.13			*r* = 0.22	*r* = 0.24	*r* = 0.31
Vietnam (Trinh et al. 2009 [12])	169 adults aged 25–64 years	14	*r* = 0.69		Moderate: *r* = 0.63; vigorous: *r* = 0.62	Moderate: *r* = 0.64	Moderate: *r* = 0.74; vigorous: *r* = 0.50	*r* = 0.69	
		Long term (two months)	*r* = 0.55		Moderate: *r* = 0.47; vigorous: *r* = 0.68	Moderate: *r* = 0.55	Moderate: *r* = 0.32; vigorous: *r* = 0.37	*r* = 0.50	
Bangladesh, Brazil, China, Ethiopia, India, Indonesia, Japan, Portugal, and South Africa (Bull et al. 2009 [1])	2657 male and female adults from nine countries.	3–7	Bangladesh			Moderate: *r* = 0.57; vigorous: *r* = 0.72; total: *r* = 0.58	*r* = 0.57	Moderate: *r* = 0.31	
			Shanghai, China			Moderate: *r* = 0.99; vigorous: *r* = 0.92; total: *r* = 0.99	*r* = 0.98	Moderate: *r* = 1.00; vigorous: *r* = 1.00; total: *r* = 1.00	
			Taiwan, China			Moderate: *r* = 0.40; vigorous: *r* = 0.48; total: *r* = 0.53	*r* = 0.54	Moderate: *r* = 0.50; vigorous: *r* = 0.49; total: *r* = 0.52	
			Ethiopia			Moderate: *r* = 0.50; vigorous: *r* = 0.64; total: *r* = 0.56	*r* = 0.53	Moderate: *r* = 0.52; vigorous: *r* = 0.46; total: *r* = 0.73	
			Indonesia			Moderate: *r* = 0.78; vigorous: *r* = 0.68; total: *r* = 0.80	*r* = 0.70	Moderate: *r* = 0.45; vigorous: *r* = 0.61; total: *r* = 0.52	
			Japan			Moderate: *r* = 0.85; vigorous: *r* = 0.88; total: *r* = 0.83	*r* = 0.90	Moderate: *r* = 0.83; vigorous: *r* = 0.89; total: *r* = 0.88	
			South Africa			Moderate: *r* = 0.75; vigorous: *r* = 0.69; total: *r* = 0.76	*r* = 0.75	Moderate: *r* = 0.77; vigorous: *r* = 0.71; total: *r* = 0.71	

Note: MET = metabolic equivalent of task; PA = physical activity.
